# Care Team Perspectives and Acceptance of Telehealth in Scaling a Home-Based Primary Care Program: Qualitative Study

**DOI:** 10.2196/12415

**Published:** 2019-06-02

**Authors:** Andrzej Kozikowski, Jillian Shotwell, Eve Wool, Jill C Slaboda, Karen A Abrashkin, Karin Rhodes, Kristofer L Smith, Renee Pekmezaris, Gregory J Norman

**Affiliations:** 1 Center for Health Innovations and Outcomes Research Northwell Health Manhasset, NY United States; 2 Northwell Health Solutions Northwell Health Manhasset, NY United States; 3 West Health Institute La Jolla, CA United States

**Keywords:** home-based primary care, homebound patients, telehealth technology

## Abstract

**Background:**

Novel and sustainable approaches to optimizing home-based primary care (HBPC) programs are needed to meet the medical needs of a growing number of homebound older adults in the United States. Telehealth may be a viable option for scaling HBPC programs.

**Objective:**

The purpose of this qualitative study was to gain insight into the perspectives of HBPC staff regarding adopting telehealth technology to increase the reach of HBPC to more homebound patients.

**Methods:**

We collected qualitative data from HBPC staff (ie, physicians, registered nurses, nurse practitioners, care managers, social workers, and medical coordinators) at a practice in the New York metropolitan area through 16 semistructured interviews and three focus groups. Data were analyzed thematically using the template analysis approach with Self-Determination Theory concepts (ie, relatedness, competence, and autonomy) as an analytical lens.

**Results:**

Four broad themes—pros and cons of scaling, technology impact on staff autonomy, technology impact on competence in providing care, and technology impact on the patient-caregiver-provider relationship—and multiple second-level themes emerged from the analysis. Staff acknowledged the need to scale the program without diminishing effective patient-centered care. Participants perceived alerts generated from patients and caregivers using telehealth as potentially increasing burden and necessitating a rapid response from an already busy staff while increasing ambiguity. However, they also noted that telehealth could increase efficiency and enable more informed care provision. Telehealth could enhance the patient-provider relationship by enabling caregivers to be an integral part of the patient’s care team. Staff members raised the concern that patients or caregivers might unnecessarily overutilize the technology, and that some home visits are more appropriate in person rather than via telehealth.

**Conclusions:**

These findings suggest the importance of considering the perspectives of medical professionals regarding telehealth adoption. A proactive approach exploring the benefits and concerns professionals perceive in the adoption of health technology within the HBPC program will hopefully facilitate the optimal integration of telehealth innovations.

## Introduction

Estimates show that there are between 1 and 4 million homebound US adults aged 65 and older [1-3]. As the senior population doubles over the next few decades, these estimates are likely to increase substantially. Homebound individuals often have complex chronic conditions and comorbidities, including heart failure, dementia, cancer, psychosocial issues, diminished functional status, and a higher risk of death [4-6]. There is increasing concern that older homebound adults are disadvantaged due to experiencing difficulties in attending traditional primary care office visits, which results in significantly decreased access to care. Instead, they rely heavily on costly emergency department visits and hospitalizations, which lead to further deterioration of health, diminished functional status, institutionalization, and a hastened death [7]. Ornstein et al found that the homebound are much more likely to have been hospitalized in the past year than their nonhomebound counterparts (52% vs 16%), and very few (12%) receive home-based primary care (HBPC) [3]. Given that long-term care and assisted-living facilities cannot accommodate the projected numbers of older patients with complex chronic conditions [8], the demand for HBPC programs will likely increase. To better address the increasing number of people needing care at home, more effective and less expensive care models are critically needed.

A recent systematic review of nine studies found that HBPC programs decrease emergency department visits, hospitalizations, and long-term care admissions while increasing patient and caregiver quality of life and satisfaction [9]. These programs improve performance of activities of daily living (eg, dressing and bathing) and instrumental activities of daily living (eg, managing medications) while reducing symptoms of depression and facilitating aging in place [10]. In one study, an HBPC program also demonstrated decreased monthly per-patient health care spending and hospital utilization [11]. However, the literature is mixed regarding the cost-effectiveness of HBPC, with some studies showing reduced costs and others demonstrating increased costs [12-16].

Telehealth, defined as the remote provision of health care through various telecommunication technologies, such as tablets, mobile phones, and other devices, is one modality that may assist in meeting these growing demands [17]. Whereas advances in medical technology in the previous century once threatened the survival of HBPC as a medical model, new mobile medical technology has facilitated the expansion of care in the home [18]. Telehealth is gaining broader acceptance and may improve the efficiency and capability of HBPC programs. Current efforts in telehealth include going beyond expanding access to care by also providing convenience to patients, expanding telehealth use from addressing acute conditions to addressing chronic conditions, and moving telehealth beyond hospitals to the home and mobile devices [17].

Care team acceptance of telehealth use is critically important, particularly in the early phases of initiating such programs [19]. It is essential to consider how telehealth is perceived by team members to influence service changes, patient-provider interactions, provider credibility, and autonomy, as well as technical issues resulting from adopting the technology [19]. Segar et al found that integrating telehealth technologies into community primary care involves adjusting provider roles and responsibilities [20]. Thus, considering the perspectives of medical professionals regarding telehealth before rather than after implementation is more likely to result in successful telehealth service integration [20].

Wade et al explored factors contributing to long-term success and sustainability of telehealth services [21]. Researchers found that clinician acceptance or willingness to either initiate or work with existing telehealth services explained the majority of the variance in telehealth uptake, enlargement, and sustainability. Clinician acceptance was a key factor for overcoming multiple barriers to success, including weak demand for telehealth, technical problems with the technology, and a paucity of funding resources. Studies of home-based telehealth for care of long-term chronic conditions have demonstrated positive outcomes, albeit many utilized poor methodological approaches and lacked theoretical frameworks [22,23]. Moreover, there is a dearth of research specifically investigating telehealth in HBPC for older adults with complex and advanced chronic conditions.

Self-Determination Theory (SDT) provides a useful theoretical framework for understanding motivations underlying the adoption of telehealth as well as, more generally, the acceptance of organizational change [24-26]. SDT proposes that people have three psychological needs: relatedness, competence, and autonomy [27-29]. Relatedness refers to the need for *belonging* and for caring relationships [27]. Competence is the need to experience mastery, and autonomy is the need to have control over choices and actions [27]. If satisfied, these needs can promote the growth of motivation. If the HBPC team perceives telehealth to foster these three psychological needs, they will be more motivated to accept and utilize it. Given that telehealth can substantially disrupt workflows, it is crucial to take into account how it may affect relatedness, competence, autonomy, and, in turn, motivation to adopt this technology to scale the HBPC program.

The primary objective of this study was to obtain greater insight into the perspectives and motivation of the HBPC team regarding the adoption of telehealth technology to scale the program to increase its capacity to reach more eligible patients in the community without adding additional care delivery team members.

## Methods

### Setting and Context

This study was conducted at a large integrated health system, which includes an HBPC program, also referred to as an Advanced Illness Management (AIM) program, consisting of interdisciplinary care teams with 11 primary care providers (ie, nurse practitioners and physicians), 9 care managers (ie, social workers and registered nurses), and 8 medical coordinators. The goal of the HBPC program is to provide longitudinal primary care to homebound, medically complex patients to meet their care needs in the home so they can remain living at home and avoid unnecessary hospital stays and emergency department visits. The care is patient centered, focusing on the patient’s goals of care, and much of the care is palliative rather than curative.

Annually, the program provides care to nearly 2000 unique individuals in Queens and Long Island, New York, NY. Those enrolled in the program are homebound; typically have multiple chronic conditions such as dementia, heart failure, and diabetes; and are in the last 1-3 years of life. The HBPC program partners with other programs within the health system along the continuum of care, such as emergency medical services, including a robust community paramedicine program [30]; home care nursing services; infusion therapy; and hospice. The HBPC program consists of interdisciplinary teams consisting of two providers, one nurse care manager, one social work care manager, and one medical coordinator.

### Design

We conducted a qualitative study using in-depth, semistructured interviews and focus groups with a purposive sample of physicians, nurse practitioners, social workers, and medical coordinators from the HBPC program. We sought to understand the perspectives of the HBPC team on adopting telehealth technology. Practice care team members were invited to volunteer to participate in the interviews and focus groups as part of ongoing process improvement activities within the program. Participants were selected to provide a cross-section of different positions within the program. Semistructured interviews and focus groups were conducted using topic guides until data saturation was attained (ie, no new topics emerged with additional interviews). The topic guides are available in Multimedia Appendix 1. Focus groups were conducted after conducting the interviews. The focus groups served to review and revisit common issues that arose during the individual interviews. One member of the research team (AK) conducted the interviews. Two members of the research team (AK and RP) facilitated the focus groups. Institutional Review Board approval was attained before study initiation. Participants were informed of the study purpose, guaranteed confidentiality, and given the right to withdraw at any time.

### Data Collection

Qualitative data were collected between February and August 2017. A total of 16 individual semistructured interviews were conducted with providers (5 physicians, 31%; 1 nurse practitioner, 6%), 4 registered nurses (25%), 3 social workers (19%), and 3 medical coordinators (19%). A total of 12 participants out of 16 were women (75%) and 4 were men (25%) (1 registered nurse, 25%; 3 physicians, 75%). Most interviews were between 15 and 30 minutes in length.

After all of the interviews were completed, three focus groups were conducted with 6-8 participants, which lasted 60-90 minutes. The first focus group included 6 administrative staff (5 female, 83%; 1 male, 17%), the second focus group included 8 care managers (4 registered nurses, 50%; 4 social workers, 50%―all female), and the third focus group consisted of 6 providers (4 physicians, 67%―2 female, 50%, 2 male, 50%; 2 female nurse practitioners, 33%). Of the 20 participants in the focus groups, half (2 administrative staff, 10%; 4 care managers, 20%; 4 providers, 20%) had also participated in the individual interviews. The moderator guides included a range of questions on perspectives regarding adopting telehealth as well as other ideas to scale the HBPC program.

Semistructured interviews and focus groups were digitally recorded, stored on an internal server to ensure security, and professionally transcribed. Transcripts were checked against the original recordings to ensure accuracy. NVivo 10 software (QSR International) was used to facilitate data storage, retrieval, and analysis.

### Data Analysis

We used the template approach to analyze, in depth, the semistructured interview and focus group transcripts [31]. First, we constructed an initial coding template containing SDT concepts (ie, relatedness, competence, and autonomy) and codes representing preliminary themes identified in the data through careful reading and review of the text. Codes were organized hierarchically so that the highest-level codes represented broad themes in the data, with lower levels indexing more narrowly focused concepts within these themes. The initial list of codes was modified through successive readings of the transcripts until we achieved as full a description of the data as was feasible.

## Results

### Overview

The central focus of the interviews and focus groups was to explore ways to scale the HBPC program using health technology (ie, different types of telehealth such as messaging services, remote monitoring, and video visits) while still maintaining the “high-touch” nature of the program. Four broad themes—pros and cons of scaling, technology impact on autonomy, technology impact on competence in providing care, and technology impact on the patient-caregiver-provider relationship—and multiple second-level themes emerged from the analysis. We present quotes from the semistructured interviews that were representative of the themes.

### Pros and Cons of Scaling

When participants were asked what the strengths of the HBPC program were, all indicated the vital service that it provides to homebound older adults and the need to expand and help more patients. The care team expressed concern and empathy for homebound patients waiting to be in the program and patients’ health conditions, as described by a social worker:

Patients will say, “I've been waiting two years to be on the program.” That's very sad. Or just to know that we can't help more people. Whenever you go out to a patient's home and you see that they've been homebound for some time with very limited support and resources, and it can be very difficult to observe and to think of the what-ifs. If only someone had gotten in sooner. So I think that's one of the biggest parts—tragedies for me, not being able to reach more people, because there's such a great need.

Social worker #1

Despite acknowledging that scaling the program is needed, concern and ambivalence was also expressed regarding increasing the patient census, as this might make it more difficult to provide the same level of personalized care. This conflict was exemplified by the same social worker who said the following:

But I think that as you grow, it takes something away...that intimacy. So it's hard. I think I'm struggling with that balance of getting bigger and having a larger census because...it'd be impossible to maintain the same type of relationship as I did when I had half the amount of people. I could see them more often. And now as we grow, we have to stretch it out more. Again, it's bittersweet for me because I like it small. But I know we have to grow...

Social worker #1

### Technology Impact on Autonomy

#### Overview

Two second-level themes were identified within this main theme: increased burden and ambiguity. Different team members indicated being extremely busy and suggested that the addition of telehealth, the influx of data from the technology, and the need to respond to the alerts would be a burden limiting their autonomy in terms of controlling decisions and actions. Specifically, for providers, differentiating urgent clinical issues from nonurgent tasks or questions was considered vital when adopting telehealth. Having someone filter the telehealth alerts before they are sent to the provider for a response was perceived as needed for this technology to work correctly and to avoid physician burnout. In addition, some care team members indicated that adopting telehealth could lead to loss of control and increased ambiguity related to when and how to monitor and respond (ie, via telehealth or the phone) to patient and caregiver communications.

#### Increased Burden

When asked about incorporating telehealth to scale the program, the following comments were given by the participants:

It may be more of a burden if I'm bombarded with alerts.

Social worker #1

Yeah. There’s a lot of—not documentation so much, but a lot of checklists, I guess that is—that are just kind of slow on the computer. But it’s not a big deal. It exists everywhere, but it’s a hassle. It’ll just be one more checklist for people to fill out. I think it would add a layer of paperwork.

Provider #1

I know that the office provider, it’s not like they ever have a free moment that they’re not on the phone. If they’re not getting a call, they’re checking the prescription line to refill the prescriptions or they’re calling a patient back that left a message. So now, they also have to check this?

Social worker #2

I think it would be a mistake to have push notifications that are unfiltered to a physician-level of care, personally. I think there has to be some kind of clinical judgment before it raises to the level of the physician or the physician's just going to get burned out. Because we're already getting so many tasks a day that the last thing we need is another ten tasks a day without it being filtered, at least.

Provider #2

I think the NP [nurse practitioner] in the office probably couldn’t watch it [telehealth alerts] because we’re already taking care of a million other things here, so I think it would be too much to add this to the NP’s responsibility in the office.

Provider #3

Because then every time you get an alert, now you have to respond to that alert. And sometimes it might just be a nonclinical issue.

Registered nurse #1

#### Ambiguity

Regarding ambiguity, the following comments were given by the participants:

And I just think that if you give people too many options, it can get confusing in a lot of ways, you know? Well, do we call that person, do we go online and contact you that way?

Social worker #2

I think it’s good, but who’s watching it? You know what I mean? Who’s—say if they—say all the sudden that the heart rate’s up or their blood pressure is up. Who’s getting the alert? You know what I mean? That’s the big thing. Who’s actually monitoring it?

Provider #3

What’s the responsibility of the provider? Because basically, at any hour in the day, any caregiver can go onto this [platform] and ask a question, and it could be a nerve-wracking question to just let lie. So what’s the responsibility?

Social worker #2

### Technology Impact on Competence in Providing Care

#### Overview

Two second-order themes were identified within this central theme: increased efficiency and more informed care provision. The HBPC team perceived telehealth to increase efficiency by decreasing the need to travel to patient homes. The technology was reported to decrease the need for community paramedics. Instead of always sending a community paramedic to measure vital sign data, remote monitoring could be conducted first to assess how the patient is doing. Participants also reported that remote monitoring would enable them to make more informed decisions about patient care.

#### Increased Efficiency

Regarding increased efficiency, participants stated the following:

It allows you to touch more people without having to do that travel time. So I think it would be a great idea. Because a lot of the time wasted really is the packing up and saying goodbye and getting into the car and driving to the next house. So what you could see in two hours, you may see two patients in two hours, while you may see four patients by video in two hours. You could see double the amount of patients without even moving. So seems like it would be more efficient.

Social worker #1

And then not have to utilize the resources of sending a paramedic into the home to check a pulse ox [oximetry] in the middle of the night. So yes, things like that I think would be the most useful, like having the ability to take a snapshot of them and send a picture and to check his—just a quick pulse ox and heart rate and possibly blood pressure.

Provider #2

#### More Informed Care Provision

Regarding more informed care provision, a provider stated the following:

Sometimes I've decided, “oh, it would be nice if I had a pulse ox...” This is just someone who's anxious and is not someone who's really in extremis. And you have to talk them through it [anxiety]. And it would be much easier for me to feel comfortable doing that if I saw a pulse ox that's 99 percent when I'm talking to them.

Provider #2

### Technology Impact on Patient-Caregiver-Provider Relationship

#### Overview

Three second-order themes were identified within this main theme: opportunity to make caregivers part of the team, overuse of technology by patients or caregivers, and some visits being more appropriate in person. Some participants felt that telehealth would enable caregivers to be part of the care team by increasing communication with the HBPC team about patient health status. However, there was also concern for caregivers and patients who might overuse the technology (eg, measure vital signs more often than needed), leading to unnecessary distress. Many team members felt that live visits should not be substituted entirely with video visits, as it is important to be present for difficult conversations physically, to convey empathy, and to perform physical exams.

#### Opportunity to Make Caregivers Part of the Team

Regarding the second-order theme, opportunity to make caregivers part of the team, a social worker stated the following:

Well, I'm trying to think what would be out of their scope. Like if they—well, let's say somebody had edema. And it was significant enough that it alarmed the HHA—the home health aide. What they are supposed to do is either call their family member and/or call their agency, right? What I'd like them to do is be able to call us, too, directly. As it stands now, my understanding is they really can't do that. But if there's a need, I want them to be able to be part of the team. Really, that's what I'm saying.

Social worker #3

#### Patients or Caregivers Might Overuse the Technology

Regarding the second-order theme, patients or caregivers might overuse the technology, a physician stated the following:

And it almost comes to the point where...they [caregiver] don’t need to be checking something multiple times a day, where they’re [patient] at a point in their life where they don’t need to check a finger stick three times a day, they don't need to check a blood pressure three times a day. And it becomes problematic because it just creates more caregiver stress that's unnecessary.

Physician #2

#### Some Visits Better in Person

Regarding the second-order theme, some visits are better in person, participants stated the following:

I wouldn’t love to do it for all my visits because a lot of my psychosocial needs or a lot of end-of-life visits or goals-of-care visits or stuff where there’s a lot of emotion, I think is really effective in person where you can use body language, and you can touch a person, you know? So I think it all—there’s a place for it, and there’s a place where I think it would actually do more harm than good.

Social worker #2

I think it has to also be looked at, the satisfaction piece. It's very hard to have people who are used to—as a nurse, there's nothing better than touching and being with them. So having field people relegated to computer-based work is tricky. And there's a level of satisfaction. I don't think the pendulum should swing completely there. I think there could be a balance. So they still have that fieldwork, and that's work that for our patients needs to get done. That's [behind a computer] not really where we solely want to go. But I do think that there is room for that kind of work. Absolutely.

Registered nurse #2

And a lot of patients felt that that was definitely still impersonal. They would rather have someone there that’s touching them and examining them and talking to them, and you know, just little things if you’re—it’s hard to convey empathy or sympathy to someone via the camera sometimes when you’re giving them bad news. And that little simple holding of the hand really goes a long way kind of thing. So a lot of the elderly patients that I’ve come across, they didn’t like that concept on video.

Provider #4

## Discussion

The primary objective of our study was to obtain greater insight into the perspectives and motivation of the HBPC team regarding adopting telehealth to scale the program. The HBPC team acknowledged the need to scale the program to help more patients but was concerned about diminishing the personalized care they provide as the census increases. Using concepts of SDT as a framework, our results showed that adopting telehealth technology is perceived as having an unfavorable impact on autonomy, particularly remote monitoring; a favorable impact on competence in providing care; and a mixed impact on the patient-caregiver-provider relationship. Participants viewed telehealth favorably to the extent that it could increase efficiency, enable more informed care provision, and facilitate caregiver involvement. Abrashkin et al, in a study with HBPC patients and caregivers, found that caregivers were more likely to have access to and feel confident in using technology such as computers, Internet, tablets, and mobile phones when compared with patients [32]. An opportunity exists to involve caregivers in the use of telehealth technology to enable them to be part of the care team.

Incorporating telehealth into the daily workload was perceived as decreasing their autonomy, given the increased burden of remote monitoring, responding to alerts, and extra paperwork for an already busy care team. They expressed concern about the potential ambiguity and confusion around controlling choices in communicating with patients (eg, online, via a telehealth app), uncertainty regarding who will be responsible for responding to alerts, and which alerts need immediate responses. Regarding telehealth’s impact on competence in providing care, participants believed that the technology could increase efficiency given the gained time not having to travel, decrease the number of community paramedic visits in which only an assessment is performed without treatments given, and increase informed care provision due to remote monitoring of patient health data. With regard to technology’s impact on the patient-caregiver-provider relationship, the HBPC team believed that telehealth could facilitate increased caregiver involvement making them part of the care team. However, many participants indicated that some patients and caregivers might overuse the technology, which will increase patient and caregiver distress waiting to hear back on the technology platform.

Additionally, some visits would not be applicable via telehealth, especially those requiring seeing patients in their context, communicating bad news, and visits requiring an in-person presence to convey empathy. Replacing such visits with video visits was consistently mentioned to impersonalize the experience, limit empathy, and decrease patient satisfaction with the care they receive.

Adopting telehealth may be a potential best practice for improving efficiency and scalability, which, in turn, may address two significant challenges for HBPC programs: that community needs often exceed program capacity and the necessity of providing urgent visits [33]. Previous research conducted in non-HBPC settings has shown that provider acceptance of telehealth is a crucial determinant of successful adoption and sustainability [19,21]. Moreover, acceptance of telehealth is often a slow process impeded by negative perceptions of the technology [34]. In a recent study assessing nursing staff facilitators and barriers to telehealth use, Koivunen et al found that nurse attitudes toward telehealth remain somewhat negative and are thus a barrier to implementation [35]. Of note, there are no previously published studies exploring HBPC team perspectives on adopting telehealth; our findings are both consistent and differ from that previously reported in the literature in non-HBPC settings.

Given the slow adoption of technology in the health care sector [34,36], it is crucial to address care team perceptions regarding telehealth [20]. The SDT concepts of relatedness, competence, and autonomy are a useful framework to assess motivation to adopt telehealth technology in scaling HBPC programs [26]. Moreover, it is important to consider different functions of telehealth [17,22]. In our study, virtual visits were perceived more favorably when compared to remote monitoring. The HBPC team were concerned about remote monitoring due to loss of autonomy having to monitor patient data and respond to alerts but saw value in virtual visits for noncritical situations. This finding reflects the direction in which our HBPC program is going, where we have initiated a telehealth implementation for virtual visits but without remote monitoring. Future research should assess these perceptions through quantitative methodology across multiple HBPC practices.

A strength of our study was the use of two types of qualitative data: semistructured interviews and focus groups. An advantage of semistructured interviews is that this enables participants to voice their own opinions and perspectives without the influence of other viewpoints. The primary advantage of conducting focus groups is that the dynamic interaction among participants can increase the depth of inquiry, stimulating discussion of experiences and their meaning to each. A combination of semistructured interviews and focus groups can yield a richer, more complex, and insightful understanding of participant perspectives [28]. However, the study also had limitations that are important to consider. It is important to note that although participants were encouraged to express their perspectives freely at all the times, some may have felt inhibited from providing views that are more critical. Because data were collected at only one HBPC practice, the findings may have limited generalizability to other practices.

In conclusion, telehealth technologies that promote HBPC team motivation are more likely to be adopted and used over time. Our findings support the importance of considering the perspectives of medical professionals regarding telehealth adoption [21]. A proactive approach exploring the benefits and concerns professionals perceive in the adoption of health technology within the HBPC program is likely to facilitate the integration of telehealth innovations.

## Appendix

Multimedia Appendix 1 Topic guides for semistructured interviews and focus groups.

## Abbreviations

AIM: Advanced Illness Management
HBPC: home-based primary care
HHA: home health aide
NP: nurse practitioner
ox: oximetry
SDT: Self-Determination Theory

## References

[1] Levine SA, Barry PP, Cassel CK, Leipzig R, Cohen HJ, Larson EB, Meier DE (2003). Home care. Geriatric Medicine: An Evidence-Based Approach. 4th edition.

[2] Qiu W, Dean M, Liu T, George L, Gann M, Cohen J, Bruce M (2010). Physical and mental health of homebound older adults: An overlooked population. J Am Geriatr Soc.

[3] Ornstein K, Leff B, Covinsky K, Ritchie C, Federman A, Roberts L, Kelley A, Siu A, Szanton S (2015). Epidemiology of the homebound population in the United States. JAMA Intern Med.

[4] McGregor M, Slater J, Sloan J, McGrail KM, Martin-Matthews A, Berg S, Plecash A, Sloss L, Trimble J, Murphy JM (2017). How's your health at home: Frail homebound patients reported health experience and outcomes. Can J Aging.

[5] Leff B, Carlson C, Saliba D, Ritchie C (2015). The invisible homebound: Setting quality-of-care standards for home-based primary and palliative care. Health Aff (Millwood).

[6] Tanner EK, Martinez IL, Harris M (2014). Examining functional and social determinants of depression in community-dwelling older adults: Implications for practice. Geriatr Nurs.

[7] Sinha S (2011). Why the elderly could bankrupt Canada and how demographic imperatives will force the redesign of acute care service delivery. Healthc Pap.

[8] Stevenson D (2008). Planning for the future: Long-term care and the 2008 election. N Engl J Med.

[9] Stall N, Nowaczynski M, Sinha S (2014). Systematic review of outcomes from home-based primary care programs for homebound older adults. J Am Geriatr Soc.

[10] Szanton S, Leff B, Wolff J, Roberts L, Gitlin L (2016). Home-based care program reduces disability and promotes aging in place. Health Aff (Millwood).

[11] Melnick G, Green L, Rich J (2016). House calls: California program for homebound patients reduces monthly spending, delivers meaningful care. Health Aff (Millwood).

[12] Beck R, Arizmendi A, Purnell C, Fultz B, Callahan C (2009). House calls for seniors: Building and sustaining a model of care for homebound seniors. J Am Geriatr Soc.

[13] Hughes S, Weaver F, Giobbie-Hurder A, Manheim L, Henderson W, Kubal J, Ulasevich A, Cummings J, Department of Veterans Affairs Cooperative Study Group on Home-Based Primary Care (2000). Effectiveness of team-managed home-based primary care: A randomized multicenter trial. JAMA.

[14] North L, Kehm L, Bent K, Hartman T (2008). Can home-based primary care cut costs?. Nurse Pract.

[15] Kinosian B, Taler G, Boling P, Gilden D, Independence at Home Learning Collaborative Writing Group (2016). Projected savings and workforce transformation from converting independence at home to a Medicare benefit. J Am Geriatr Soc.

[16] De Jonge E, Jamshed N, Gilden D, Kubisiak J, Bruce S, Taler G (2014). Effects of home-based primary care on Medicare costs in high-risk elders. J Am Geriatr Soc.

[17] Dorsey E, Topol E (2016). State of telehealth. N Engl J Med.

[18] Kao H, Conant R, Soriano T, McCormick W (2009). The past, present, and future of house calls. Clin Geriatr Med.

[19] Brewster L, Mountain G, Wessels B, Kelly C, Hawley M (2014). Factors affecting front line staff acceptance of telehealth technologies: A mixed-method systematic review. J Adv Nurs.

[20] Segar J, Rogers A, Salisbury C, Thomas C (2013). Roles and identities in transition: Boundaries of work and inter-professional relationships at the interface between telehealth and primary care. Health Soc Care Community.

[21] Wade V, Eliott J, Hiller J (2014). Clinician acceptance is the key factor for sustainable telehealth services. Qual Health Res.

[22] Wootton R (2012). Twenty years of telemedicine in chronic disease management: An evidence synthesis. J Telemed Telecare.

[23] Hanlon P, Daines L, Campbell C, McKinstry B, Weller D, Pinnock H (2017). Telehealth interventions to support self-management of long-term conditions: A systematic metareview of diabetes, heart failure, asthma, chronic obstructive pulmonary disease, and cancer. J Med Internet Res.

[24] Deci EL, Ryan RM (2000). The "what" and "why" of goal pursuits: Human needs and the self-determination of behavior. Psychol Inq.

[25] Dewar A, Bull T, Malvey D, Szalma J (2017). Developing a measure of engagement with telehealth systems: The mHealth Technology Engagement Index. J Telemed Telecare.

[26] Gagné M, Koestner R, Zuckerman M (2000). Facilitating acceptance of organizational change: The importance of self‐determination. J Appl Soc Psychol.

[27] Deci EL, Ryan RM, Van Lange PAM, Kruglanski AW, Higgins ET (2012). Self-determination theory. Handbook of Theories of Social Psychology: Volume One.

[28] Chirkov V, Ryan R, Kim Y, Kaplan U (2003). Differentiating autonomy from individualism and independence: A self-determination theory perspective on internalization of cultural orientations and well-being. J Pers Soc Psychol.

[29] Deci EL, Vansteenkiste M (2004). Self-determination theory and basic need satisfaction: Understanding human development in positive psychology. Ric Psicol.

[30] Abrashkin K, Washko J, Zhang J, Poku A, Kim H, Smith K (2016). Providing acute care at home: Community paramedics enhance an advanced illness management program-Preliminary data. J Am Geriatr Soc.

[31] Symon G, Cassell C (1998). Qualitative Methods and Analysis in Organizational Research: A Practical Guide.

[32] Abrashkin K, Patel V, Kozikowski A, Zhang M, Poku A, Pekmezaris R (2018). Access to and confidence in using technology among homebound older adults and caregivers. J Am Med Dir Assoc.

[33] Norman G, Orton K, Wade A, Morris A, Slaboda J (2018). Operation and challenges of home-based medical practices in the US: Findings from six aggregated case studies. BMC Health Serv Res.

[34] Taylor J, Coates E, Brewster L, Mountain G, Wessels B, Hawley M (2015). Examining the use of telehealth in community nursing: Identifying the factors affecting frontline staff acceptance and telehealth adoption. J Adv Nurs.

[35] Koivunen M, Saranto K (2018). Nursing professionals' experiences of the facilitators and barriers to the use of telehealth applications: A systematic review of qualitative studies. Scand J Caring Sci.

[36] Kane C, Gillis K (2018). The use of telemedicine by physicians: Still the exception rather than the rule. Health Aff (Millwood).

