# Supplementary material for: Care Team Perspectives and Acceptance of Telehealth in Scaling a Home-Based Primary Care Program: Qualitative Study
Source: JMIR Aging. 2019 Jun 2;2(1):e12415. doi: 10.2196/12415 (PMC6716443; doi:10.2196/12415)
Supplement: Multimedia Appendix 1 [file aging_v2i1e12415_app1.pdf]

APPENDIX

House Calls Staff Semi-Structured Interview Topic Guide

Participant's ID: __________

Participant Position: ☐ Physician ☐ Nurse Practitioner ☐ Registered Nurse ☐ Social Worker

☐ Care Coordinator ☐ Other: Please specify: __________________

Date of Interview: __________

Initials of Interviewer: _______

[SAY] Thank you so much for agreeing to be interviewed for this project.

As part of the West Health Grant, we are seeking to optimize the function of the House Calls program so that we can increase the number of patients you serve. To do this, we're hoping to learn more from you about your thoughts and experiences working at House Calls. Thank you for agreeing to participate, as a reminder this is completely optional and confidential; nobody who supervises you will see any of your answers. All names will be removed, and results are only reported as a group, not individually. Are you okay to begin the interview?

1. Overall, please tell me about your experience caring for patients in the *House Calls* program.

    a. Probe: What about *House Calls* is most beneficial/meaningful/comforting to patients/caregivers?

    b. Probe: What are the strengths of the *House Calls program*?

    c. Probe: What are the weaknesses of the *House Calls program*?

    d. Probe: What would you change to make *House Calls* better?

    e. Probe: Did you experience any barriers to providing good quality care for patients in *House Calls?* What were the barriers?

    f. Probe: How do you feel about the number of times per month *House Calls* staff visited the patient's home? Should it remain the same/increase/decrease? Why? What would be the optimal number of visits per month? Why?

2. How do you feel about increasing patient census? Why?

3. If you could have anything you needed, how would you design *House Calls* to make it more efficient?

Now let's talk about changing the House Calls program by adding telehealth technology. This would consist of the patient and/or caregiver using a computer based communication tool to share vital signs data which would allow us to monitor patient health status remotely.

4. How would you feel if we added telehealth technology in the patient's home as part of their care in the *House Calls* program?

    a. Probe: Would adding this technology make you feel comfortable/uncomfortable? Why?

    b. Probe: Would it be helpful/unhelpful? Why?

    c. Probe: Would this technology make your work more efficient? Why or why not?

    d. Probe: Would there be any barriers for you to use this technology? What are they? How comfortable are you with telehealth technology? Do you have any experience with telehealth? Can you describe?

    e. Would the health monitoring technology make caring for the patient easier/more difficult? Why?

5. How would you/the provider feel if we substituted some of the live visits with video visits?

6. Do you have any other recommendations about the *House Calls* program?

7. Do you have any questions/comments about what we had talked about today?

<u>Comments</u>: INTERVIEWER, use this space to summarize how the interview went, including the mood, facial expressions and body language of the participant during the interview.

House Calls Staff Focus Group Topic Guide

Introduction: House Calls is a much-needed program that takes excellent care of the most vulnerable patients near the end of their life.

However, the program is currently not financially sustainable. One of the ways to improve financial sustainability is to see more patients more efficiently.

The goal of the West Health project is to address this problem and see how we can scale the program, without compromising the quality of care and maybe even serve as a national model for how best to provide home-based primary care sustainably.

1. Because you are on the front lines and know the program the best, we need your perspective on the optimal way to do this.

What are your thoughts on how we can best scale the program?

- a. **Probes:**
  - i. Barriers
  - ii. Facilitators
  - iii. Suggestions made in the interviews:
  - iv. What are other ideas not mentioned that could help scale the program?

2. Another area we wish to explore is whether or not health IT could help us scale the program by improving communication between patients/caregivers and the house calls care team.

In general, what kinds of approaches would be helpful from the health IT perspective to make your job easier to help your patients?

- a. **Probes:**
  - i. One example of health IT that staff suggested may be useful were video visits
    1. Advantages/disadvantages/frequency
  - ii. Another example of health IT that staff suggested may be useful was HIPPA secure text messaging
    1. Advantages/disadvantages/frequency
  - iii. Are there other ways health IT could help?
  - iv. What work flows would need to be changed if we integrated health IT? *(probe by profession)*
  - v. How do we incorporate increased flexibility into the home visits for care managers/providers?
  - *vi.* What screening mechanisms should we put in place for acute calls
  - vii. Provider desk
